# Exploiting machine-learning models with DCE-MRI radiomics for breast cancer molecular subtyping

**DOI:** 10.3389/fonc.2026.1794638

**Published:** 2026-08-10

**Authors:** Yang Lou, Liang Zhao

**Affiliations:** 1 Department of Radiology, Affiliated Hospital of Hebei University, Baoding, Hebei, China; 2 Hebei Key Laboratory of Precise Imaging of Inflammation-Related Tumors, Affiliated Hospital of Hebei University, Baoding, Hebei, China

**Keywords:** breast cancer molecular subtyping, DCE-MRI–based radiomics model, feature selection, multi-class classification model, ROI feature extraction

## Abstract

Breast cancer is a heterogeneous disease, and accurate preoperative identification of molecular subtypes is essential for guiding individualized treatment and prognostic evaluation. However, histopathological assessment, the current gold standard, is invasive and limited by intratumoral heterogeneity, underscoring the need for reliable noninvasive alternatives. Radiomics based on dynamic contrast-enhanced magnetic resonance imaging (DCE-MRI) has shown promise for molecular subtype prediction; nevertheless, the optimal radiomic features for automated molecular classification remain to be fully elucidated. To address this gap, this study aims to identify optimal DCE-MRI radiomic features and to develop radiomics-based machine learning models for predicting breast cancer molecular subtypes. Specifically, regions of interest were manually delineated on DCE-MRI images for feature extraction, followed by feature selection to identify the most informative radiomic parameters. Four machine learning classifiers (RF, SVM, LR, GBDT) were then constructed to predict five molecular subtypes of breast cancer. The experimental results demonstrate that the optimized DCE-MRI–based radiomics model effectively predicts breast cancer molecular subtypes. In particular, the gradient boosting decision tree (GBDT) model combined with rigorous feature selection shows high predictive performance, highlighting its strong potential for noninvasive, accurate, and clinically applicable molecular classification of breast cancer.

## 1 Introduction

Breast cancer is the most commonly diagnosed malignancy among women and remains the fifth leading cause of cancer-related mortality worldwide, with approximately 2.3 million new cases diagnosed globally in 2020 Sung et al. (1). Despite substantial advances in screening programs and individualized therapeutic strategies that have improved patient survival, the absolute number of breast cancer deaths worldwide was projected to increase from 561,334 in 2015 to 805,116 by 2030, representing an estimated 43% increase Cardoso et al. (2). This highlights the persistent clinical and public health burden of the disease. Breast cancer is a biologically heterogeneous disease. According to the St. Gallen International Breast Cancer Expert Consensus Conference, it is classified into four major molecular subtypes based on immunohistochemical characteristics: luminal A, luminal B, human epidermal growth factor receptor 2 (HER2)-enriched, and triple-negative breast cancer (TNBC) Goldhirsch et al. (3). These molecular subtypes are closely associated with tumor behavior, treatment response, and prognosis, and therefore play a central role in guiding clinical management. Luminal A tumors generally show favorable outcomes, with good responses to endocrine therapy and low risks of recurrence and metastasis. In contrast, luminal B tumors exhibit higher proliferative activity and an increased risk of early recurrence, despite their sensitivity to endocrine treatment. HER2-enriched and TNBC subtypes are typically more aggressive and associated with poorer prognoses; however, patients with HER2-enriched tumors can benefit significantly from targeted anti-HER2 therapies Sun et al. (4). Consequently, accurate preoperative biological characterization of breast cancer is essential for optimizing personalized treatment strategies and improving patient outcomes.

Currently, early breast cancer diagnosis relies on radiological evaluation followed by histopathological confirmation via biopsy. Although biopsy remains the clinical gold standard, it is invasive and may be limited by intratumoral heterogeneity, restricted accessibility in certain settings, and procedure-related risks. Accordingly, imaging modalities such as mammography, ultrasound, and magnetic resonance imaging (MRI) have become essential for tumor detection and subtype assessment. Among these, MRI is particularly valuable because it is noninvasive, provides high spatial resolution without ionizing radiation, and offers both morphological and functional information, thereby improving diagnostic accuracy Neeter et al. (5)Heck and Herzen (6)Savaridas and Tennant (7). Consequently, MRI has been widely adopted for breast cancer evaluation in clinical practice. Emerging evidence indicates that breast cancer molecular subtypes are associated with distinct radiological phenotypes. Luminal tumors often demonstrate irregular morphology, spiculated margins, and heterogeneous enhancement on MRI Seyfettin et al. (8). HER2-overexpressing tumors more frequently present with irregular or lobulated shape, multifocal lesions, and segmental non-mass enhancement accompanied by prominent vascularity Grimm et al. (9)An et al. (10)Huang et al. (11). TNBC commonly appears as a round or oval mass (often larger than 2 cm) with marked rim enhancement, consistent with its aggressive biology and rapid growth Huang et al. (11)Sung et al. (12). Differences have also been reported in enhancement kinetics, with luminal A tumors more often showing Type II curves, whereas luminal B, HER2-enriched, and TNBC subtypes predominantly demonstrate Type III curves Long et al. (13). However, these associations are largely qualitative and depend on subjective interpretation, which may not fully capture subtle heterogeneity. Accordingly, imaging-based attempts to predict molecular subtypes using conventional qualitative features have shown suboptimal and inconsistent performance, often due to limited feature representation and incomplete subgroup characterization. This underscores the need for a more comprehensive and quantitative assessment of MRI-derived information.

Radiomics provides such a quantitative framework by extracting high-dimensional, handcrafted features that describe tumor intensity distributions, shape, and texture patterns, thereby offering complementary information beyond visual assessment. In principle, radiomics can noninvasively infer underlying molecular characteristics and support personalized decision-making. Nevertheless, clinical translation remains challenging because radiomics analyses can be sensitive to imaging protocol variability, feature redundancy, and overfitting in high-dimensional settings; moreover, limited standardization and external validation often restrict generalizability. Radiomics approaches typically include traditional machine-learning pipelines based on handcrafted features as well as deep learning–based methods that learn hierarchical representations directly from images; both have shown promise for malignancy characterization, treatment response prediction, and molecular profiling. Among MRI sequences, dynamic contrast-enhanced MRI (DCE-MRI) is particularly informative because it captures lesion morphology, perfusion, vascular permeability, and enhancement kinetics, which are imaging surrogates of tumor microenvironment and intratumoral heterogeneity. Recent studies have shown that DCE-MRI and multiparametric MRI radiomics can support the preoperative assessment of lymphovascular invasion, HER2 expression patterns, histological grade, treatment response, and other biologically relevant breast cancer characteristics by extracting quantitative information from post-contrast enhancement patterns Yang et al. (14, 15); Zheng et al. (16)Shang et al. (17)Chen et al. (18)Dai et al. (19)Wang et al. (20)Li et al. (21). Compared with qualitative visual assessment, DCE-MRI radiomics therefore provides a reproducible feature space for modeling subtle phenotype differences among breast cancer subtypes. However, existing MRI-based radiomics studies have often focused on selected subtype comparisons, biomarker-specific binary classification, or simplified subtype schemes. In addition, many models rely on a large number of features, which can increase complexity and limit interpretability and generalizability. These limitations highlight the need for an efficient and robust radiomics framework capable of comprehensive multiclass molecular subtype prediction, while also enabling clinically meaningful refinement within heterogeneous categories such as luminal B disease.

To address these gaps, we develop and evaluate a DCE-MRI radiomics–based multiclass machine-learning framework for breast cancer molecular subtyping. Specifically, we aim to: (i) construct a feature-selection pipeline to reduce redundancy and identify informative radiomic predictors from DCE-MRI; (ii) develop and compare multiple machine-learning classifiers for multiclass prediction, including a five-class scheme that distinguishes luminal B HER2-negative from luminal B HER2-positive disease; and (iii) assess performance on an independent held-out test cohort while highlighting a compact set of key radiomic features to improve interpretability and potential clinical applicability. In a retrospective cohort of 505 patients, our optimized DCE-MRI radiomics model demonstrated effective multiclass molecular subtype prediction. Notably, gradient boosting decision trees achieved the best discrimination, with an AUC of 0.99 in the training set and 0.83 in the independent test set. Moreover, rigorous feature selection yielded a compact radiomics signature, supporting interpretability and clinical feasibility. Collectively, these results suggest that a carefully selected DCE-MRI radiomics signature combined with a robust machine-learning classifier may provide a promising noninvasive tool for preoperative breast cancer molecular stratification. The main contributions of this study are as follows:

- We formulate breast cancer molecular subtype prediction as a multiclass task using DCE-MRI radiomics, and adopt a five-class scheme that further separates luminal B HER2-negative from luminal B HER2-positive disease.
- We construct a compact radiomics signature via a two-stage feature-selection strategy, enhancing interpretability and robustness.
- The proposed noninvasive framework offers potential support for treatment planning and prognostic evaluation by enabling preoperative molecular stratification.

## 2 Materials and methods

This section describes the study design to develop and evaluate a DCE-MRI radiomics model for breast cancer molecular subtype prediction. Specifically, we detail patient enrollment with inclusion/exclusion criteria and ethical approval, the MRI acquisition protocol on two 1.5-T scanners, and the pathological reference standard for molecular subtype assignment based on IHC/FISH following the 2013 St. Gallen consensus. We further outline the radiomics pipeline, including ROI delineation and quality control, image preprocessing (voxel resampling, intensity normalization, and gray-level discretization), feature extraction and selection, stratified data partitioning, machine-learning model construction (SVM, RF, LR, and GBDT), class-imbalance handling, and performance evaluation using ROC-derived AUC and overall accuracy.

### 2.1 Study design and patient selection

This retrospective study consecutively screened 520 patients with breast cancer between January 1, 2019 and December 1, 2024. Patients were eligible if they met all of the following criteria:

- histopathologically confirmed invasive breast cancer (biopsy or surgical specimen) with complete immunohistochemical data enabling molecular subtype assignment.
- preoperative breast MRI performed within 7 days before surgery.
- availability of key clinical variables, including age and menopausal status.

Patients were excluded if:

- They had received percutaneous biopsy, neoadjuvant chemotherapy, or radiotherapy prior to MRI.
- They had lesions that could not be reliably evaluated due to severe artifacts or the absence of a definable region of interest (ROI).
- They had poor-quality or incomplete MRI data.
- They had missing immunohistochemical information required for molecular typing.

After applying these criteria, 505 patients were included for subsequent radiomics analyses.

### 2.2 MRI equipment and image acquisition

Breast MRI examinations were performed with patients in the prone position using dedicated breast surface coils on two 1.5-T MRI systems (Siemens and United Imaging Healthcare [UIH]). The institutional protocol included axial T2-weighted imaging, axial T1-weighted imaging, diffusion-weighted imaging (DWI), and axial dynamic contrast-enhanced (DCE) imaging. For the DCE sequence acquired on the Siemens system, parameters were: repetition time (TR) 4.53 ms, echo time (TE) 1.75 ms, slice thickness 1.5 mm, field of view (FOV) 340 mm × 340 mm, flip angle 10°, and matrix 384 × 384. For the UIH system, DCE parameters were: TR 5.12 ms, TE 2.32 ms, slice thickness 2.0 mm, FOV 320 mm × 320 mm, flip angle 10°, and matrix 291 × 416. A contrast agent was administered via an antecubital vein using a power injector at a flow rate of 2 mL/s and a dose of 0.2–0.3 mmol/kg. After injection, 5–6 consecutive dynamic acquisitions without inter-scan intervals were obtained, with each acquisition lasting approximately 60–90 s.

Because images were acquired from two scanners with slightly different spatial resolutions, all subsequent radiomics analyses used standardized resampling, intensity normalization, and gray-level discretization to reduce scanner- and protocol-related variability before feature extraction.

### 2.3 Pathological analysis and molecular subtype definition

Molecular subtypes were determined according to the expert consensus of the 2013 St. Gallen International Breast Cancer Conference, based on immunohistochemistry (IHC) and fluorescence *in situ* hybridization (FISH) results in postoperative pathological reports. ER and PR positivity were defined as nuclear staining in ≥ 1% of tumor cells. HER2 positivity was defined as an IHC score of 3+ or an IHC score of 2+ with HER2 gene amplification confirmed by FISH Wolff et al. (22). Ki-67 positivity was defined as an expression level of ≥ 14%. Based on these criteria, tumors were categorized into five molecular subtypes: (1) TNBC (triple-negative breast cancer; ER−/PR−, HER2−), (2) HER2-enriched (ER−/PR−, HER2+), (3) Luminal A (ER/PR+, HER2−, low Ki-67), (4) Luminal B (HER2+) (ER/PR+, HER2+), and (5) Luminal B (HER2−) (ER/PR+, HER2−, high Ki-67).

### 2.4 Radiomics data processing

Radiomics data processing included the following three steps: (1) ROI segmentation and quality control, in which tumors were manually delineated on DCE-MRI to generate lesion masks and then reviewed by a senior radiologist; (2) Image preprocessing and radiomics feature extraction, including voxel resampling, z-score intensity normalization, gray-level discretization/binning, and extraction of shape, first-order, and texture features; and (3) Feature selection, where redundant features were removed and LASSO was applied within the training cohort to identify informative features that were subsequently used to train supervised classifiers for molecular subtype prediction.

#### 2.4.1 ROI segmentation

**Figure 1:**
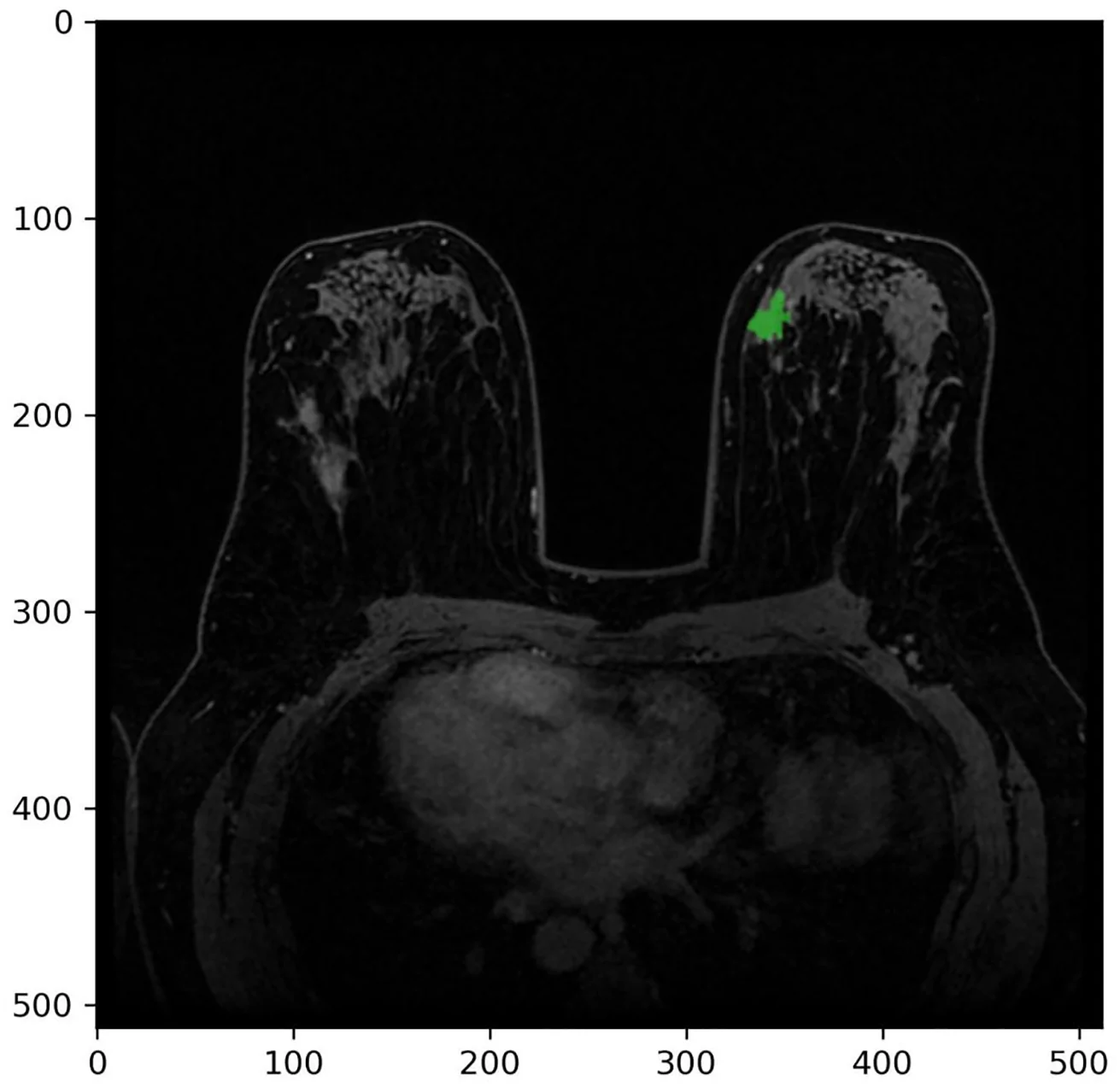
Representative tumor segmentation on axial DCE-MRI. The green overlay denotes the manually delineated ROI used for radiomics feature extraction.

For image segmentation, data were retrieved from the PACS system and stored in DICOM format. The region of interest (ROI) was contoured on the axial DCE images using ITK-SNAP software (version 3.8.0). ROI delineation followed predefined criteria: (1) the lesion was outlined on the phase showing the clearest enhancement, with reference to other sequences when necessary to better identify lesion boundaries; (2) contours were drawn slice by slice to include as much tumor parenchyma as possible, including necrotic or cystic components when present, so that intratumoral heterogeneity could be represented; (3) peripheral vessels and adjacent non-tumor tissues were excluded; and (4) all ROIs were delineated by radiologists experienced in breast MRI and then reviewed by a senior radiologist. Disagreements were resolved through consensus discussion and contour adjustment. An example of an ROI profile is presented in **Figure 1**.

#### 2.4.2 ROI quality control and feature robustness

Radiomics features can be sensitive to segmentation variability. To reduce this source of variation, all lesion masks were generated under the same ROI protocol and underwent senior-radiologist review before feature extraction. The final analysis used consensus ROIs, which ensured consistency of the masks used for model construction. However, because repeated independent contours were not available in the archived dataset, formal intra- and interobserver intra-class correlation coefficient (ICC) analysis could not be retrospectively calculated. Consensus review reduces gross contouring errors but does not replace quantitative segmentation-reproducibility testing. This limitation is explicitly acknowledged in the Discussion, and future prospective work should include repeated segmentation by at least two independent readers on a representative subset of cases, followed by ICC-based feature robustness screening before model training.

#### 2.4.3 Image preprocessing and feature extraction

To improve the robustness and reproducibility of radiomics features across different scanners and acquisition protocols, all DCE-MRI images and their corresponding region-of-interest (ROI) masks were preprocessed using a standardized pipeline before feature extraction. The DCE series used for segmentation was resampled to an isotropic voxel spacing of 1 × 1 × 1 mm^3^ via linear interpolation to harmonize spatial resolution across subjects. The associated binary ROI masks were resampled to the same spacing using nearest-neighbor interpolation to preserve label integrity and prevent boundary smoothing artifacts. After resampling, intensity normalization was applied to reduce inter-scan signal scale differences. For each case, voxel intensities within the ROI were normalized using z-score standardization. Let *X*_orig_ denote the original voxel intensity, and let *µ*_ROI_ and *σ*_ROI_ represent the mean and standard deviation of intensities computed within the ROI, respectively. The normalized intensity *X*_norm_ was defined as:

(1) $$X_{\text{norm}}=\frac{X_{\text{orig}}-\mu_{ROI}}{\sigma_{ROI}}.$$

This notation distinguishes the original voxel intensity from the normalized value and avoids using the same symbol for both quantities. Prior to texture feature calculation, gray-level discretization was performed to reduce noise-related intensity fluctuations and to ensure comparable texture computation across patients.

Radiomics features were then extracted in 3D using SimpleITK and PyRadiomics, including shape, first-order statistics, and texture features. Thus, for the *i*-th subject, the feature vector is denoted as

$xi\;\in \;{\mathbb{R}}^{p}$, and the feature matrix is

(2) $$X={\left[x_{1},x_{2},\ldots ,x_{n}\right]}^{⊤}\in {\mathbb{R}}^{n\times p},$$

where *n* is the number of samples.

#### 2.4.4 Feature selection

To reduce feature redundancy and mitigate overfitting, a two-stage feature selection strategy was implemented using the training cohort only. First, all radiomics features were standardized by z-score normalization, and pairwise correlation analysis was performed to identify highly collinear variables. For any feature pair with an absolute correlation coefficient greater than 0.95, one feature was removed to eliminate redundant information and improve model stability. No information from the held-out test cohort was used during this process. Second, the remaining features were further screened using the least absolute shrinkage and selection operator (LASSO) Tibshirani (23), which imposes an **L**_1_-norm penalty to shrink less informative coefficients toward zero and thereby performs embedded feature selection. The LASSO optimization problem is defined as:

(3) $$\hat{\beta }=\text{arg }\underset{\beta }{\min}\{\frac{1}{2n}\sum_{i=1}^{n}{\left(y_{i}-\beta_{0}-x_{i}^{T}\beta \right)}^{2}+\lambda {\mathrm{||\beta ||}}_{1}\},$$

where n denotes the number of samples, xi is the radiomics feature vector of the i-th sample, and yi is the corresponding label. Here, β0 denotes the intercept, β represents the regression coefficients, and λ controls the strength of sparsity enforced by the ℓ1-penalty $∥\beta {∥}_{1}=\sum_{j=1}^{p}|\beta_{j}|$. In this study, LASSO was applied to the training set with *λ* searched over the range [0.01,100] using cross-validation, a tolerance of 1 × 10^−5^, and a maximum of 200 iterations. The optimal regularization parameter was *α* = 0.106, which retained three non-zero radiomics features while preserving model performance. These features were then used for model construction and final evaluation on the independent test set.

### 2.5 Reporting standards and reproducibility considerations

The radiomics workflow was evaluated with reference to the Image Biomarker Standardisation Initiative (IBSI), the Radiomics Quality Score (RQS), and the Checklist for Artificial Intelligence in Medical Imaging (CLAIM) recommendations Zwanenburg et al. (24)Lambin et al. (25)Mongan et al. (26). To enhance methodological transparency and reproducibility, we provide detailed descriptions of the image-acquisition protocol, region-of-interest delineation and review procedures, image resampling, intensity normalization, gray-level discretization, extracted feature families, feature-selection strategy, separation of the training and test cohorts, modeling algorithms, and performance-evaluation metrics. Nevertheless, because this study was conducted retrospectively at a single institution using privacy-protected clinical imaging data, several desirable reporting and validation practices could not be fully implemented, including prospective protocol registration, public release of case-level data, external multicenter validation, and quantitative assessment of intra- and interobserver reproducibility using intraclass correlation coefficients. These limitations are explicitly acknowledged and will inform the design of future prospective and multicenter validation studies.

### 2.6 Cohort description and data split

Following the predefined patient selection and data processing workflow, a total of 505 patients with pathologically confirmed breast cancer were included in the final analysis, comprising 504 women and 1 man. The overall mean age was 54 ± 12 years. The cohort consisted of five molecular subtypes: Luminal A (*n* = 124, 25%; mean age 55 ± 13 years), Luminal B (HER2−) (*n* = 136, 27%; mean age 54 ± 12 years), Luminal B (HER2+) (*n* = 73, 14%; mean age 52 ± 12 years), triple-negative breast cancer (TNBC) (*n* = 85, mean age 54 ± 9 years), and HER2-enriched (*n* = 87, mean age 54 ± 11 years).

With respect to clinical characteristics, 290/505 (57.4%) patients were postmenopausal and 215/505 (42.6%) were premenopausal. Most patients reported a reproductive history (490/505, 97.0%), whereas 15/505 (3.0%) did not. A family history of cancer was present in 121/505 (24.0%) patients, while 384/505 (76.0%) reported no family history. Comorbidities were noted in 243/505 (48.1%) patients and were absent in 262/505 (51.9%). Detailed distributions of menopausal status, fertility history, family history of cancer, and personal medical history across molecular subtypes are summarized in **Table 1**. To address the unequal sample sizes among molecular subtypes, the dataset was divided into training and independent test cohorts using stratified random sampling at an 8:2 ratio. Stratification preserved the subtype proportions in both cohorts and reduced the risk that minority subtypes would be under-represented in the test set. The resulting training cohort included 403 patients and the independent test cohort included 102 patients, with subtype-specific distributions shown in **Table 2**.

**Table 1:** General clinical features of the study cohort.

Molecular subtypes	Luminal A (25%)	HER2– (27%)	HER2+ (14%)	TNBC (17%)	HER2-enriched (17%)	Total
Total	124	136	73	85	87	505
Male	1	0	0	0	0	1
Female	123	136	73	85	87	504
Age (years)	55.45	53.82	51.88	53.86	53.70	53.88
Menopause(Y)	67	73	36	56	58	290
Menopause(N)	57	63	37	29	29	215
Childbearing history(Y)	121	130	71	84	84	490
Childbearing history(N)	3	6	2	1	3	15
Family history of cancer(Y)	25	40	17	25	14	121
Family history of cancer(N)	99	96	56	60	73	384
Personal medical history(Y)	51	65	36	47	44	243
Personal medical history(N)	73	71	37	38	43	262

**Table 2:** Stratified training and testing distribution by molecular subtype.

Molecular subtype	Total	Training set	Testing set
Luminal A	124	99	25
Luminal B (HER2−)	136	109	27
Luminal B (HER2+)	73	58	15
TNBC	85	68	17
HER2-enriched	87	69	18
Total	505	403	102

### 2.7 Model and evaluation

To model and discover the relationship between DCE-MRI radiomics features and breast cancer molecular subtypes, four commonly used supervised learning classifiers were employed: support vector machine (SVM) Cortes and Vapnik (27), logistic regression (LR) Cox (28), random forest (RF) Breiman (29), and gradient boosting decision tree (GBDT) Friedman (30). All classifiers were trained using only the selected radiomics features from the training cohort, and their generalization performance was evaluated on the independent test set.

#### 2.7.1 Support vector machine

Support Vector Machine (SVM) is a margin-based classifier that learns an optimal separating hyperplane by maximizing the margin between classes. Since radiomics features are typically high-dimensional and may exhibit non-linear separability, we adopted an SVM with a Gaussian radial basis function (RBF) kernel to implicitly map features into a higher-dimensional space. The RBF kernel is defined as

(4) $$K(x,x^{\prime })=\text{exp}(-\gamma ∥x-x^{\prime }{∥}_{2}^{2}),$$

where γ controls the kernel width. Two key hyperparameters were tuned: the penalty parameter *C*, which controls the trade-off between margin maximization and misclassification, and the kernel parameter *γ*, which determines the influence range of a single training sample. We performed a grid search to identify the optimal configuration, obtaining *C* = 0.280 and *γ* = 0.150. Model outputs were computed from the distance to the separating hyperplane (i.e., the decision function), which provides a continuous score for ROC/AUC analysis.

#### 2.7.2 Logistic regression

Logistic Regression (LR) is a probabilistic linear classifier that models the conditional probability of class membership as a function of a linear combination of input features. For multi-class prediction, LR estimates class probabilities via the softmax function and assigns the class with the maximum posterior probability. The model is expressed as

(5) $$P(y=k|x)=\frac{\text{exp}(w_{k}^{⊤}x+b_{k})}{\sum_{j}\text{exp}(w_{j}^{⊤}x+b_{j})},$$

where **w***_k_* and *b_k_* denote the weight vector and bias term for class *k*, respectively. LR was implemented using a linear solver, making it computationally efficient and well-suited as a strong linear baseline. The predicted class probabilities were directly used as continuous scores for ROC/AUC computation.

#### 2.7.3 Random forest

RF is an ensemble learning method that combines multiple decision trees trained on bootstrapped samples of the training data. At each split, a random subset of features is considered, which decorrelates trees and reduces variance. The final prediction is obtained by aggregating the predictions of all trees (majority vote for class labels and averaged class probabilities for scoring). RF is particularly suitable for radiomics because it can capture non-linear interactions among features and is relatively robust to feature scaling. In this study, the number of trees (estimators) was set to 10, while other parameters were kept at their default settings unless otherwise specified.

#### 2.7.4 Gradient Boosting Decision Tree

Gradient Boosting Decision Tree (GBDT) is a boosting-based ensemble method that builds an additive model by sequentially fitting decision trees to correct the errors (residuals) of the current ensemble. Formally, the model is constructed in a stage-wise manner as

(6) $$F_{m}\left(x\right)\;=F_{m}{-}_{1}\left(x\right)\;+\nu \;h_{m}\left(x\right)$$

where *h_m_*(·) denotes the *m*-th weak learner (decision tree) and *ν* ∈ (0,1] is the shrinkage factor (learning rate). At each iteration, *h_m_*(·) is trained to approximate the negative gradient of a differentiable loss function with respect to the current model output, enabling GBDT to capture complex non-linear decision boundaries and feature interactions. In this study, the number of estimators was set to 10. The predicted class probabilities (or equivalent continuous scores) from the boosted ensemble were used for ROC/AUC evaluation.

### 2.8 Evaluation metric

Receiver operating characteristic (ROC) curves were used to evaluate the discriminative ability of the classifiers by illustrating the trade-off between the true positive rate (TPR) and the false positive rate (FPR) across different decision thresholds. The TPR (also referred to as sensitivity or recall) and the FPR were defined as:

(7) $$\text{TPR}=\frac{\text{TP}}{\text{TP}+\text{FN}},$$

(8) $$\text{FPR}=\frac{\text{FP}}{\text{FP}+\text{TN}},$$

where TP (true positive) denotes the number of samples that are truly positive and correctly classified as positive, FP (false positive) denotes the number of samples that are truly negative but incorrectly classified as positive, FN (false negative) denotes the number of samples that are truly positive but incorrectly classified as negative, and TN (true negative) denotes the number of samples that are truly negative and correctly classified as negative. The area under the ROC curve (AUC) was calculated as a threshold-independent summary metric, with larger AUC values indicating better overall classification performance. In addition to AUC, overall accuracy was reported to summarize the proportion of correctly classified samples among all samples:

(9) $$\text{Accuracy}=\frac{N_{\text{correct}}}{N},$$

where *N*_correct_ denotes the number of correctly classified cases and *N* denotes the total number of cases. For the five-class molecular subtype task, ROC analysis was performed using a one-vs-rest strategy for each subtype, and the macro-level AUC was used to compare classifiers. Because this is a single-label multi-class classification task, micro-averaged precision, recall, and F1-score are mathematically equivalent to overall accuracy; therefore, they were not presented as separate columns to avoid redundant metrics. Subtype-level AUCs were also reported to show whether specific molecular subtypes were more difficult to discriminate.

## 3 Experimental results

**Figure 2:**
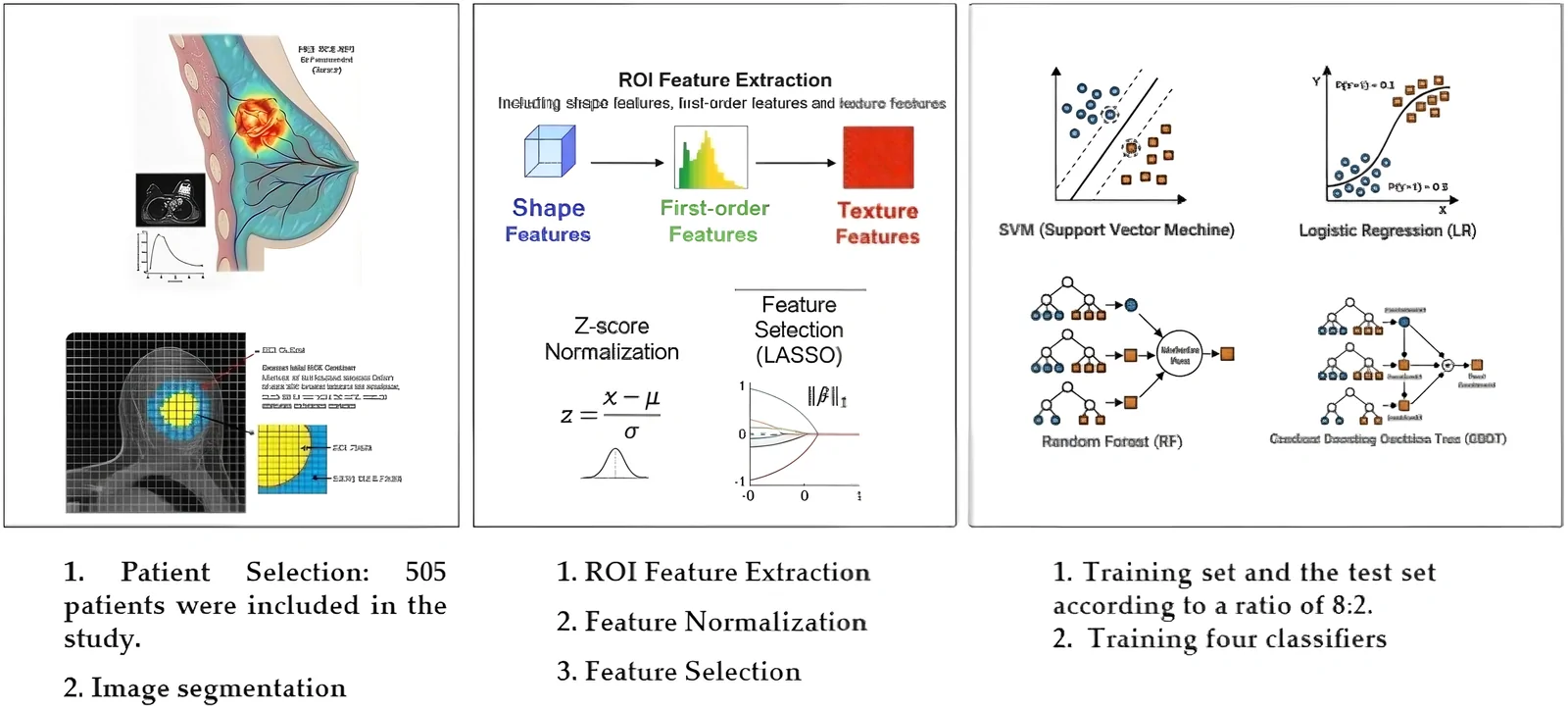
Overview of the DCE-MRI radiomics workflow. Of 520 screened breast cancer patients, 505 met the inclusion criteria and were divided into stratified training and independent testing cohorts at an 8:2 ratio. Tumor ROIs were segmented on DCE-MRI, radiomics features were extracted and selected, and four machine-learning classifiers were trained to predict five molecular subtypes.

Radiomics features were extracted from the DCE-MRI images and then reduced using the proposed two-stage feature selection strategy, which was designed to remove redundant variables and retain the most informative features for molecular subtype prediction. Feature selection was performed using the training cohort only to avoid information leakage, and the final selected feature subset was subsequently used as the common input for model development. A total of 505 patients were divided by stratified random sampling into a training set (n = 403) and an independent test set (n = 102) at an 8:2 ratio, enabling an assessment of model generalizability while preserving subtype distributions across cohorts. An overview of the entire pipeline is summarized in **Figure 2**.

### 3.1 Classification results

Four supervised learning algorithms were trained and compared, including support vector machine (SVM), random forest (RF), logistic regression (LR), and gradient boosting decision tree (GBDT). Model discrimination was primarily quantified using ROC analysis and the area under the ROC curve (AUC).

**Figure 3:**
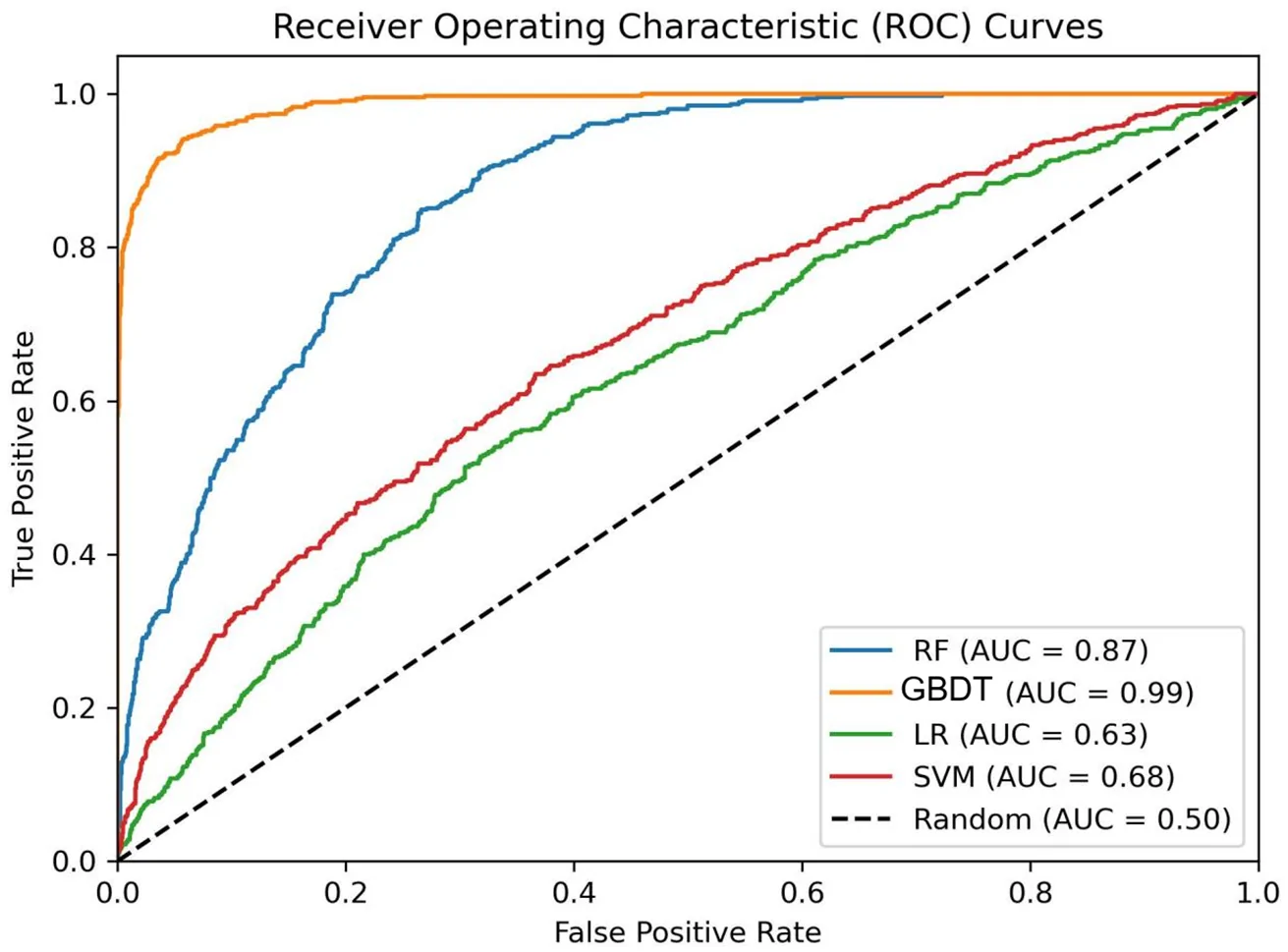
Training-set ROC curves and AUC values for the four classifiers (GBDT, LR, RF, and SVM) in the five-class molecular subtype prediction task.

**Figure 4:**
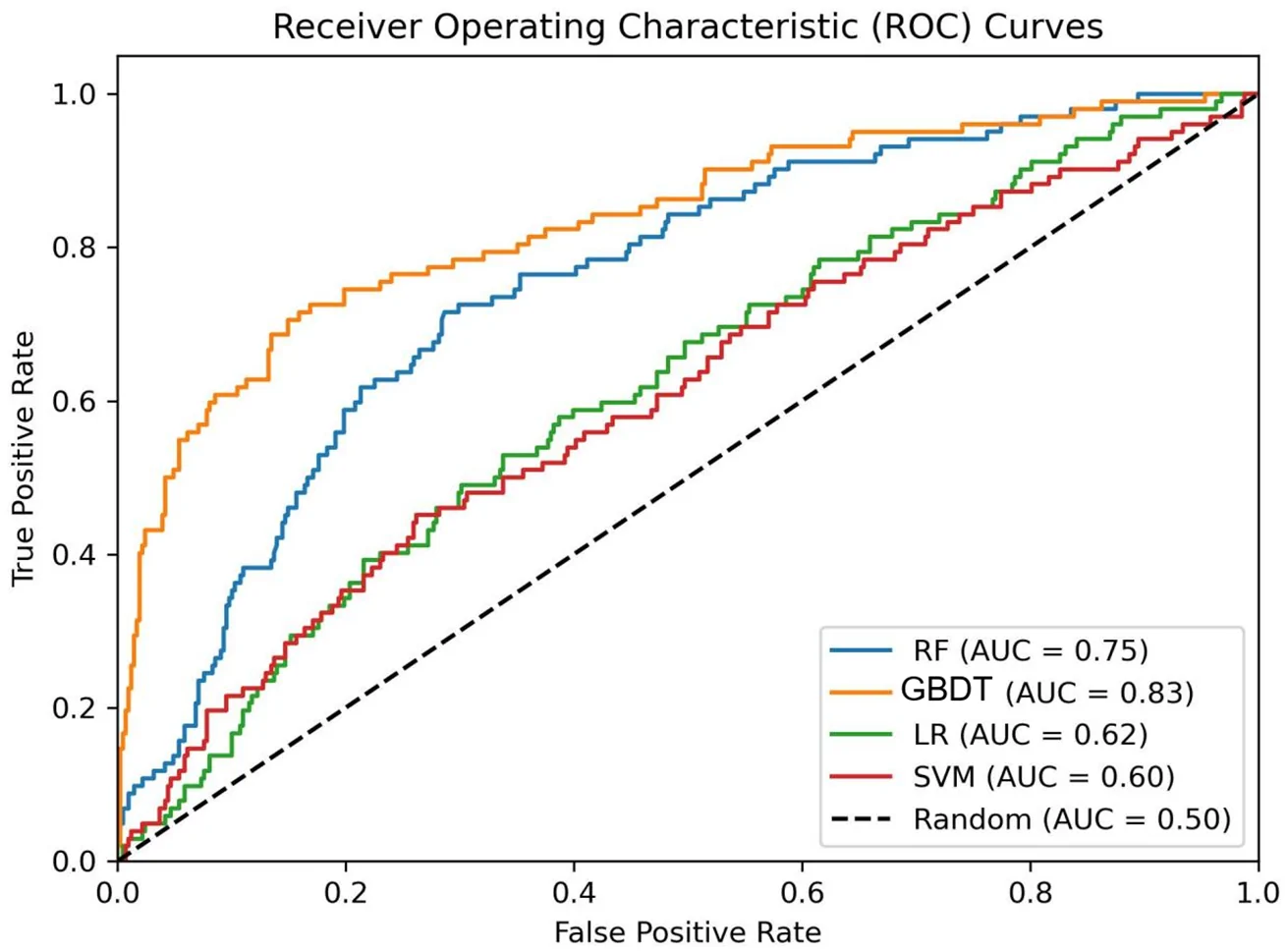
Testing-set ROC curves and AUC values for the four classifiers (GBDT, LR, RF, and SVM), showing internal-validation performance on the held-out cohort.

In the training set, GBDT demonstrated near-optimal discrimination (AUC = 0.99), and maintained the best performance in the independent test set (AUC = 0.83), indicating superior generalization compared with the other classifiers (**Figures 3**, **4**). In contrast, RF, LR, and SVM yielded lower AUCs in both cohorts: in the training set, their AUCs were 0.87, 0.63, and 0.68, respectively, while in the test set, the AUCs decreased to 0.75, 0.62, and 0.60, respectively (**Figures 3**, **4**). These findings suggest that the boosting-based ensemble model better captured the complex and potentially non-linear relationships between DCE-derived radiomics patterns and molecular subtypes. Beyond ROC/AUC, overall accuracy was reported as an intuitive summary of classification correctness. Because micro-precision, micro-recall, and micro-F1 are mathematically equivalent to accuracy in single-label multi-class classification, these redundant metrics were not listed separately. Consistent with the AUC results, GBDT achieved the highest overall performance, with an accuracy of 0.90 in the training set and 0.63 in the test set (**Table 3**). By comparison, the accuracies of LR, RF, and SVM were 0.32, 0.55, and 0.41 in the training set, and 0.28, 0.41 and 0.36 in the test set, respectively (**Table 3**). Detailed AUC and accuracy measures for all classifiers in both cohorts are provided in **Table 3**.

**Table 3:** Overall AUC and accuracy of each model in the training and testing sets.

Dataset	Method	AUC	Accuracy
Training set	GBDT	0.99	0.90
	LR	0.63	0.32
	RF	0.87	0.55
	SVM	0.68	0.41
Testing set	GBDT	0.83	0.63
	LR	0.62	0.28
	RF	0.75	0.41
	SVM	0.60	0.36

**Figure 5:**
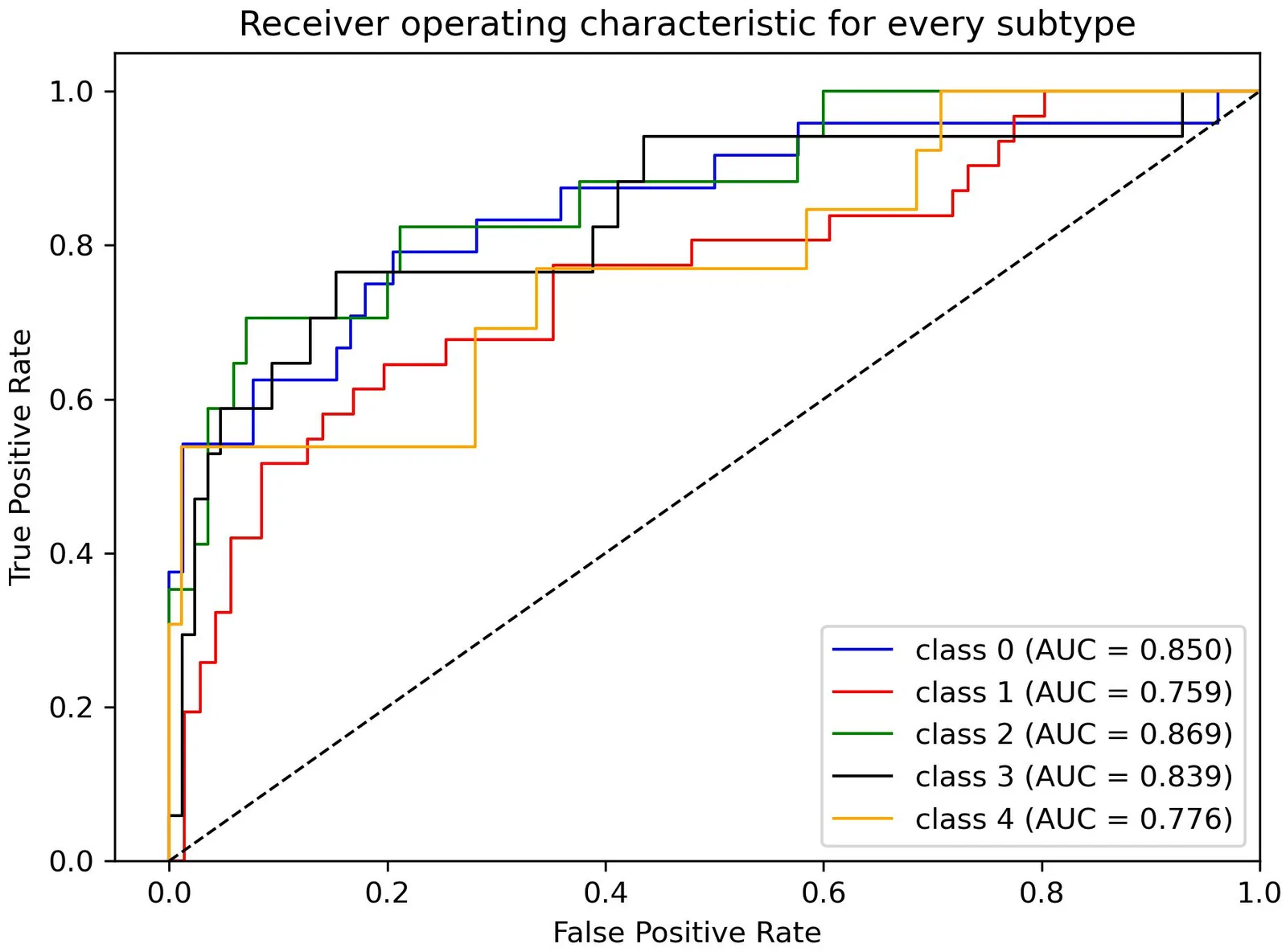
One-vs-rest testing-set ROC curves of the GBDT model for each molecular subtype. Class labels are: 0, Luminal A; 1, Luminal B (HER2−); 2, HER2-enriched; 3, TNBC; and 4, Luminal B (HER2+).

In addition to the overall multi-class performance, we further examined the one-vs-rest ROC curves for each molecular subtype using the GBDT method (**Figure 5**). The per-class AUCs ranged from 0.759 to 0.869, indicating heterogeneous discriminative difficulty across subtypes. Specifically, the model achieved the highest AUC for HER2-enriched disease (AUC = 0.869), followed by Luminal A (AUC = 0.850) and TNBC (AUC = 0.839), suggesting good separability for these categories. In contrast, Luminal B (HER2+) (AUC = 0.776) and Luminal B (HER2−) (AUC = 0.759) showed comparatively lower AUCs, implying greater overlap with other subtypes and consequently reduced classification confidence. To make subtype-level performance explicit, the testing-set one-vs-rest AUCs are summarized in **Table 4**. Overall, all subtype-specific ROC curves lay above the diagonal reference line, demonstrating that the model provided meaningful discrimination beyond chance level for all five classes.

**Table 4:** Subtype-specific one-vs-rest AUCs of the GBDT model in the testing set.

Molecular subtype	Testing samples	AUC
Luminal A	25	0.850
Luminal B (HER2−)	27	0.759
HER2-enriched	18	0.869
TNBC	17	0.839
Luminal B (HER2+)	15	0.776

### 3.2 Feature selection results

**Figure 6:**
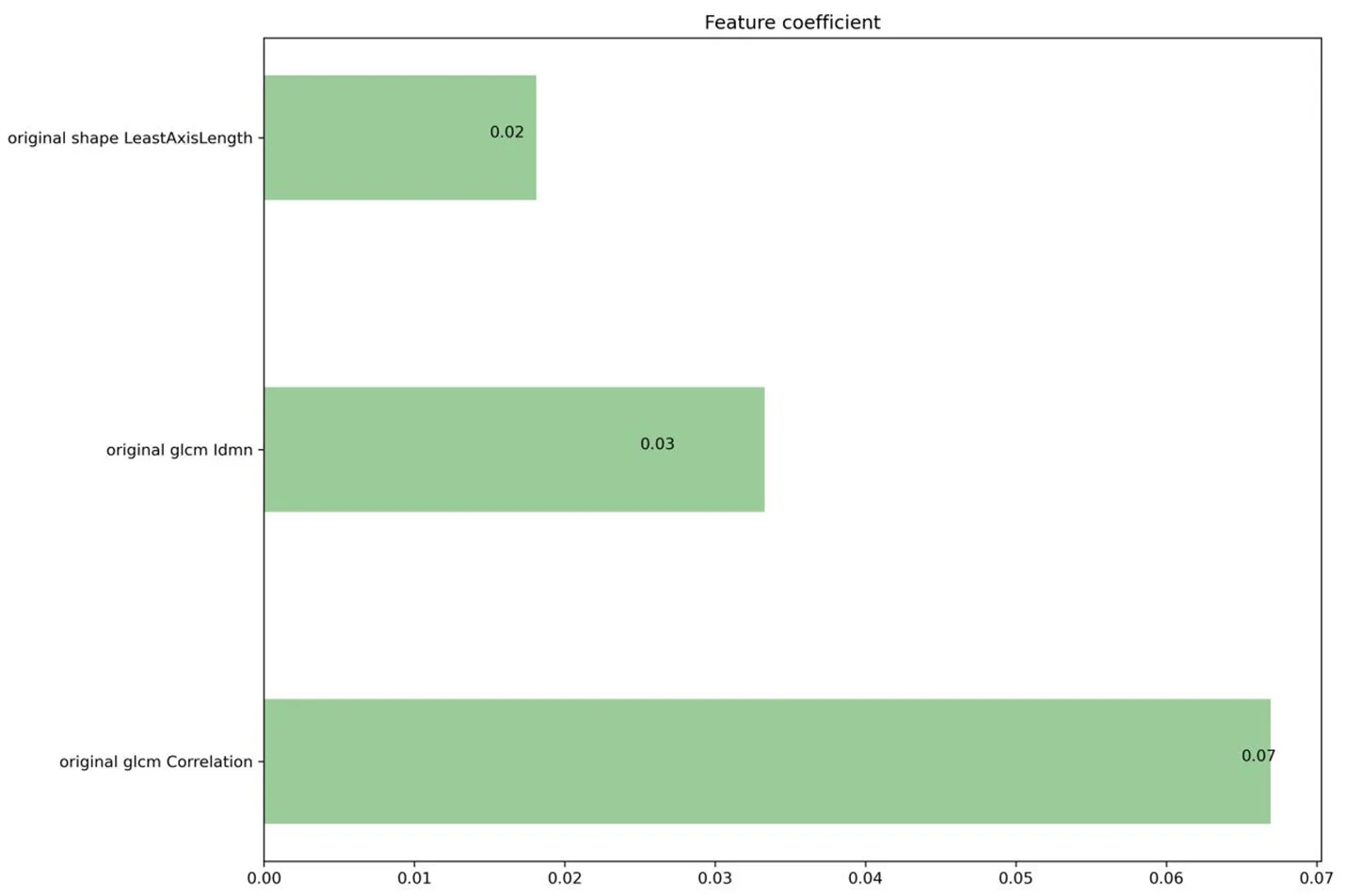
Non-zero LASSO coefficients of the final DCE-MRI radiomics signature. The x-axis lists the retained feature names, and the y-axis shows the corresponding feature coefficients.

**Figure 7:**
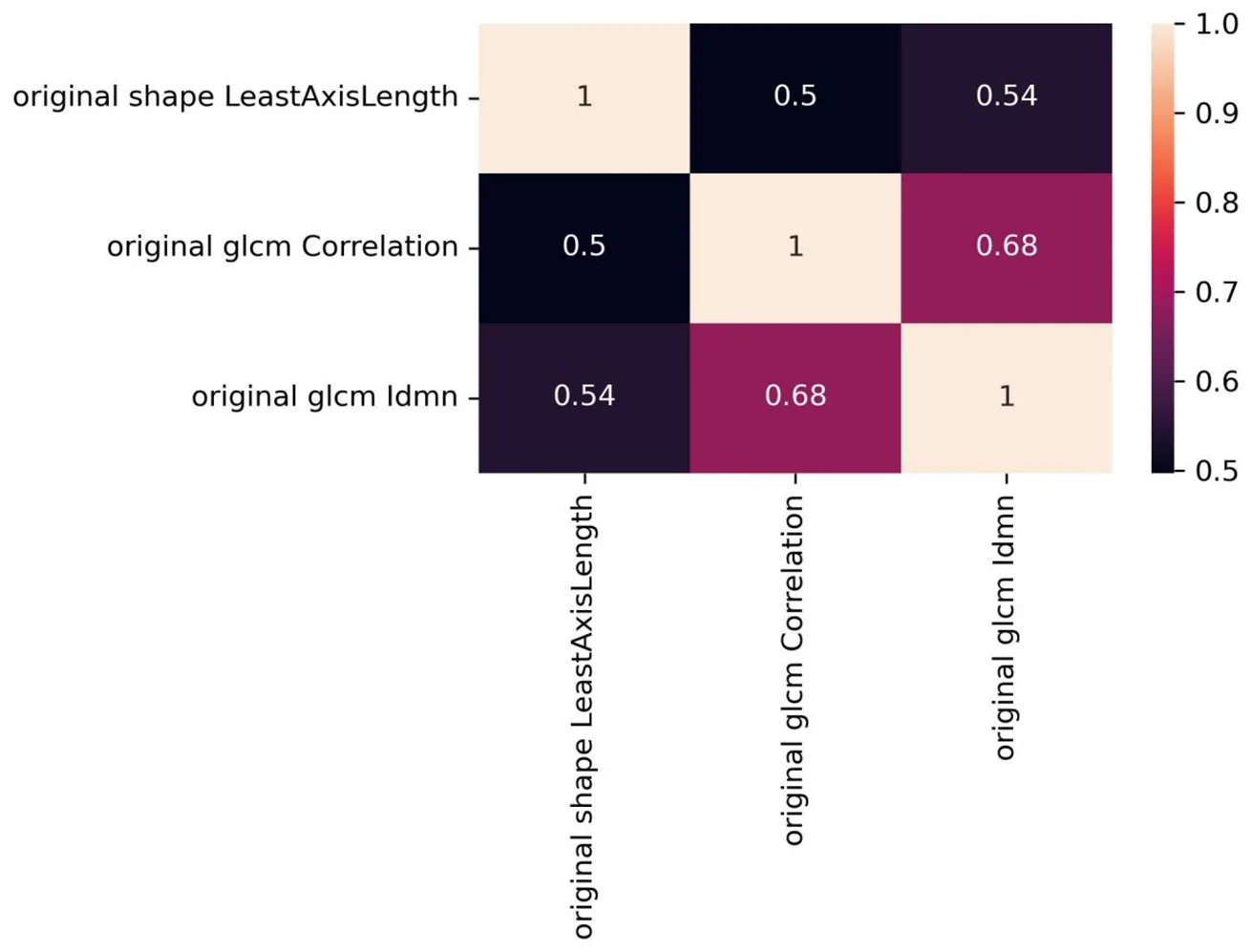
Correlation heatmap of the three retained radiomics features, used to assess residual collinearity after feature selection.

A total of 107 radiomics features were initially extracted from the DCE-MRI images. Feature selection was performed only in the training cohort. In the first stage, z-score normalization and pairwise correlation filtering were used to remove redundant highly correlated variables (absolute correlation coefficient *>* 0.95). In the second stage, LASSO was applied to the remaining candidates, with the regularization strength selected by cross-validation. Under the optimal parameter *α* = 0.106, three non-zero and non-redundant features were retained for model training, as shown in **Figure 6**. Correlation heatmap analysis indicated that these three selected features were largely independent, with no apparent multicollinearity as shown in **Figure 7**. The retained features were: original shape LeastAxisLength, original glcm Correlation, and original glcm Idmn. Specifically, original shape LeastAxisLength reflects lesion morphology/size characteristics, whereas original glcm Correlation and original glcm Idmn (inverse difference moment normalized) quantify intratumoral texture patterns and gray-level homogeneity, respectively. Feature importance analysis demonstrated that original glcm Correlation contributed most to molecular subtype discrimination, followed by original shape LeastAxisLength and original glcm Idmn. This compact feature set reduced model complexity and improved interpretability, although prospective studies with repeated ROI delineation are still needed to quantify segmentation robustness. Collectively, these features exhibited meaningful associations with breast cancer molecular subtypes, supporting their use as a compact radiomics signature for molecular typing.

## 4 Discussion and analysis

In this study, we developed four DCE-MRI radiomics models using four machine-learning algorithms: Random Forest (RF), Support Vector Machine (SVM), Logistic Regression (LR), and Gradient Boosting Decision Tree (GBDT). All models were designed to predict breast cancer molecular subtypes. After optimized feature selection and dimensionality reduction, the GBDT-based model achieved the best predictive performance, indicating that radiomics models built on traditional machine-learning algorithms remain valuable and have strong potential for molecular subtype prediction.

We further identified three key radiomics features that were critical for distinguishing among the five molecular subtypes. The strongest association was observed for the gray-level co-occurrence matrix (GLCM)–derived feature, highlighting the importance of texture information in subtype discrimination. Texture features reflect intratumoral heterogeneity. Specifically, the original GLCM Correlation quantifies the linear dependency of gray levels between neighboring pixels: higher values indicate more uniform textures, whereas lower values suggest greater heterogeneity. In addition, the original GLCM Inverse Difference Moment Normalized (IDMN) measures local homogeneity by assessing the probability of similar gray-level pixel pairs; larger values indicate more homogeneous texture. The prominence of these two texture features is consistent with previous radiomics studies Holli-Helenius et al. (31)Waugh et al. (32) Wu et al. (33) and supports the hypothesis that more aggressive tumors, such as HER2-positive disease and TNBC, exhibit higher heterogeneity on imaging.

This interpretation also aligns with known MRI manifestations and biological mechanisms. TNBC frequently demonstrates round or oval morphology and rim enhancement patterns on DCE-MRI Nguyen et al. (34), which may reflect peripheral angiogenesis and central necrosis, and is associated with increased vascular endothelial growth factor (VEGF) expression and the absence of estrogen and progesterone receptors. HER2-positive tumors often show more heterogeneous enhancement and may present with necrotic or cystic components Huang et al. (11), likely due to rapid proliferation and insufficient nutrient supply leading to necrosis. Mechanistically, amplification and overexpression of the HER2 gene enhance proliferative and survival signaling, and HER2 expression has been associated with increased VEGF expression and angiogenesis. Uneven angiogenesis can in turn lead to heterogeneous perfusion and enhancement within the tumor. Similarly, luminal B tumors exhibited greater heterogeneity than luminal A tumors in our cohort, consistent with the findings of Lafcı et al. Lafcı et al. (35). From an imaging perspective, luminal B tumors are more likely to demonstrate non-uniform enhancement, multifocal or multicentric disease, skin or nipple–areolar involvement, and axillary findings compared with luminal A, which may reflect neovascularization and/or necrosis and has been linked to biological differences such as growth factor receptor activity. Song et al. Song et al. (36) also reported the utility of related radiomics features for differentiating HER2-positive from HER2-negative disease.

In addition to texture features, the shape feature Least Axis Length also contributed substantially to subtype classification, ranking second in importance. Least Axis Length describes lesion morphology and represents the shortest principal axis of the segmented region (in 2D, the shortest axis of the fitted ellipse; in 3D, the shortest principal dimension of the fitted ellipsoid). This finding is consistent with evidence that lesion size and morphology correlate with molecular subtype. Prior studies have shown that ER-positive and PR-positive tumors tend to be smaller than receptor-negative tumors Huang et al. (11). TNBC is often larger at diagnosis than non-TNBC tumors on MRI Chen et al. (37)Liu et al. (38), and HER2-positive tumors may also be larger than other subtypes Szep et al. (39).

MRI, particularly DCE-MRI, is widely used in clinical practice because of its high sensitivity for breast cancer detection. DCE-MRI provides rich information on lesion morphology, enhancement patterns and perfusion characteristics, offering indirect insight into tumor biology and vascular supply. Although enhancement patterns differ across molecular subtypes, standardized qualitative criteria remain limited due to sample constraints and reader dependency. Radiomics can complement qualitative assessment by extracting multi-scale quantitative information that is not discernible to the naked eye, enabling objective characterization of heterogeneity and reducing subjective bias. Recent MRI radiomics studies have further extended this approach to HER2-zero/low/positive differentiation, HER2 2+ status prediction, histological grading, and DCE-MRI-derived assessment of axillary lymph node metastasis, supporting the continued relevance of breast MRI radiomics while also emphasizing the need for transparent reporting and validation Chen et al. (18)Dai et al. (19)Wang et al. (20)Li et al. (21)Liu et al. (40). Consequently, DCE-MRI has become a widely used sequence in AI-based breast cancer research and often demonstrates strong diagnostic performance.

This study has several limitations. First, no external validation was performed. Accordingly, the testing cohort should be regarded as an internally held-out test set rather than a true external validation cohort. Although the use of an independent test split provides preliminary evidence of model performance, the observed results may still be influenced by institution-specific imaging protocols, patient characteristics, scanner configurations, and segmentation practices. Future prospective studies using independent multicenter cohorts with heterogeneous scanners and acquisition protocols are therefore warranted to confirm the generalizability and clinical applicability of the proposed model. Second, although image preprocessing was performed to mitigate scanner- and protocol-related variability, the retrospective design involving two scanners may still have introduced residual scanner heterogeneity. Such heterogeneity could affect radiomic feature distributions and, consequently, model performance. Future studies should further evaluate scanner effects and consider harmonization strategies to improve model robustness across imaging platforms. Third, the reproducibility of the extracted radiomic features was not quantitatively assessed using intraclass correlation coefficients. The absence of intra- and interobserver reproducibility analyses represents an additional limitation, particularly because manual ROI delineation may introduce observer-dependent variability. Future prospective studies should include repeated ROI delineation by multiple radiologists and perform ICC-based feature robustness screening before feature selection and model development.

Overall, our findings support the feasibility of a compact DCE-MRI radiomics signature combined with GBDT modeling for noninvasive prediction of breast cancer molecular subtypes. With external validation and multi-parametric/clinical integration, this approach may contribute to preoperative risk stratification and individualized treatment planning.

## 5 Conclusions

In conclusion, this study demonstrates the potential of traditional machine-learning algorithms combined with DCE-MRI radiomics features for breast cancer molecular subtype classification. After two-stage feature selection, a compact three-feature signature was retained, and the GBDT model achieved the best overall performance among the evaluated classifiers. The model showed meaningful discrimination across all five molecular subtypes, although the two luminal B subgroups remained more difficult to separate, likely because of overlapping imaging phenotypes and subtype imbalance. However, the current testing cohort represents internal validation only, and the findings require confirmation in independent multicenter cohorts before the model can be considered clinically generalizable. Further validation should include heterogeneous scanners and acquisition protocols, harmonized preprocessing, repeated ROI delineation, multiparametric MRI, clinical feature integration, and predefined model-evaluation procedures before clinical translation.

## Data Availability

The datasets presented in this article are not readily available because they contain privacy-protected clinical imaging data. Requests to access the datasets should be directed to 18732110969@sina.cn.
